# Predictors of high out-of-pocket healthcare expenditure: an analysis using Bangladesh household income and expenditure survey, 2010

**DOI:** 10.1186/s12913-017-2047-0

**Published:** 2017-01-31

**Authors:** Azaher Ali Molla, Chunhuei Chi, Alicia Lorena Núñez Mondaca

**Affiliations:** 1 Oregon State University, 3405 NW Orchard Avenue, Apt. 106, Corvallis, OR 97330 USA; 2 Institute of Health Economics, University of Dhaka, Dhaka, Bangladesh; 3 International Health Program, School of Biological and Population Health Sciences, Oregon State University, Corvallis, OR 97330 USA; 4 Public Health, Department of Management Control and Information Systems, University of Chile, Av. Diagonal Paraguay 257, Santiago, 8330015 Chile

**Keywords:** Predictors of healthcare expenditure, Out-of-pocket expenditure, Health systems finance, Health policy, Household financial burden, Chronic illness, Rural-urban differentials

## Abstract

**Background:**

Predictors of high out-of-pocket household healthcare expenditure are essential for creating effective health system finance policy. In Bangladesh, 63.3% of health expenditure is out-of-pocket and born by households. It is imperative to know what determines household health expenditure. This study aims to investigate the predicting factors of high out-of-pocket household healthcare expenditure targeting to put forward policy recommendations on equity in financial burden.

**Methods:**

Bangladesh household income and expenditure survey 2010 provides data for this study. Predictors of high out-of-pocket household healthcare expenditure were analyzed using multiple linear regressions. We have modeled non-linear relationship using logarithmic form of linear regression. Heteroscedasticity and multicollinearity were checked using Breusch-Pagan/Cook-Weishberg and VIF tests. Normality of the residuals was checked using Kernel density curve. We applied required adjustment for survey data, so that standard errors and parameters estimation are valid.

**Results:**

Presence of chronic disease and household income were found to be the most influential and statistically significant (*p* < 0.001) predictors of high household healthcare expenditure. Households in rural areas spend 7% less than urban dwellers. The results show that a 100% increase in female members in a family leads to a 2% decrease in household health expenditure. Household income, health shocks in families, and family size are other statistically significant predictors of household healthcare expenditure. Proportion of elderly and under-five members in the family show some positive influence on health expenditure, though statistically nonsignificant.

**Conclusions:**

The findings call for emphasizing prevention of chronic diseases, as it is a strong predictor of household health expenditure. Innovative insurance scheme needs to be devised to prevent household from being impoverished due to health shocks in the family. Policy makers are urged to design an alternative source of healthcare financing in Bangladesh to minimize the burden of high OOP healthcare expenditure.

## Background

Good health of a population is an important input for poverty reduction, economic growth and long term economic development [1]. Well-planned health systems finance protects population against the financial risks with ill-health [2]. Now-a-days, healthcare costs are rising at a rate faster than any previous time because of increased aging population, more prevalence of chronic diseases, and availability of more technically sophisticated costly treatments. Out-of-pocket (OOP) payments or household’s share of direct healthcare expenditure is a major component of health system finance in middle and low-income countries. The threat that OOP payments pose to household living standards is increasingly recognized as a major consideration in health system financing [1, 3]. There is a growing evidence that household being pushed into poverty or forced into deeper poverty when faced with substantial medical expenses [4].

Out-of-pocket payments are primary means of financing healthcare in much of Asia, where the ratio of OOP payments to total household health care expenditure ranges from 30 to 82% [5], and outlays the major source of health system finance, and is notably burdensome for poor households [6]. In most of these countries, OOP expenditure for healthcare are regressive while social assistance and fee exemptions are either non-existence or where exist, are not well targeted at the most in need [7]. Bangladesh, China, India, and Vietnam stand out in relying heavily on OOP financing, having a high prevalence of catastrophic payments leading to poverty. The overall prevalence of absolute poverty in 11 Southeast Asian countries is 14% higher than conventional estimates of poverty that do not consider OOP payments for healthcare [5].

Bangladesh is one of the poorest countries in the world with per capita GDP of US $ 747 · 34 and a life expectancy at birth of 70 years in 2012 [8]. Per capita total expenditure on health is US $67 in 2011, and total expenditure on health as percentage of gross domestic product (GDP) is 3 · 7% [9]. The main source of finance for total health expenditure is OOP by household (63.3%) followed by government spending (26%) and external resource (8%) [10]. Being one of the lower-middle income countries with a population of 160 million (July 2014 estimated) [11], Bangladesh has been striving to improve its population’s health since long. On average household spends 11% of their total household budget on health and half of the residents spend 7% of their monthly per capita consumption expenditure on illness [12]. Demographic characteristics as well as severity of illness play an important role in health spending [13]. Health insurance coverage, particularly in rural areas is non-existence or if exists, remains very low. Although, public funding is negligible (US $3 · 0 out of US$11 · 0), 60–70% of which spends on essential services packages (ESPs). While ESP is helping to target resources at priority services, considerable barriers to access by vulnerable groups persist [14]. There is a large reduction in household resources associated with maternal illness, driven almost entirely by spending on healthcare [15].

Information on household healthcare expenditure is essential for creating effective health system finance policy for any country, irrespective of state of development. This information is much more crucial for middle and low-income countries. Appropriate and adequate community and country bound health policy cannot be devised without adequate knowledge of health expenditure at household level. Akanda & Minowa [16] emphasized the importance of analyses of demand for healthcare and healthcare expenditure at household level. The question that bears the burden of healthcare expenditure is a conventional question of equity, and for promoting equitable financial burden in healthcare. This study considers the importance of household health expenditure analysis on health policy formulation. Although need is a perceived phenomenon, the most obvious factor that predicts households’ OOP healthcare payments is presence of illness [17]. Among the non-need or predisposing factors, income is treated as one of the most important predictors [18]. You and Kobayashi [19] found that people spent more on healthcare with increasing age (over 65 years), chronic disease, higher incomes, and residence in urban areas. Having health insurance household increases the utilization of health care, and at the same time, decreases the amount of OOP payments. However, in Bangladesh, voluntary health insurance is nearly absent or present in some pocket areas. Bangladesh National Health accounts [10] reports that voluntary health insurance contributes 0.1% to the national health accounts.

A limited number of studies have been conducted in Bangladesh on this issue. A study on determinants of household healthcare expenditure in Chittagong Division showed that income has a significant effect on peoples’ choice of healthcare provider and on the amount of healthcare expenditure [20]. Another study showed that illness is but one of the many factors involved in utilization for healthcare. Household characteristics, educational level, type of medical consultants, location, and wealth variable significantly influence the level of healthcare expenditure [21]. The limited number of studies that conducted in these countries has several limitations. These studies mainly use income data with a very limited amount of asset data. Additionally, to establish a causal relationship, a limited number of studies use modern econometric techniques of analysis. On top of that, those studies were conducted in some pocket areas that do not represent the nation. Generalizability is essential for formulation of health system financing policy. This study uses a nation-wide household income and expenditure survey conducted in 2010. The findings of the study will be useful to device national healthcare financing policy. The primary objective of this study is to investigate the factors predicting high household expenditure incurred on healthcare in Bangladesh. The specific research questions are:

1. What are the effects of chronic illnesses and health shocks in the family on household healthcare expenditure?
2. Does rural-urban differential play any role on household healthcare expenditure in Bangladesh? and
3. Which individual household factors, i.e. age, gender, education, and family size best describes household health spending?

## Methods

### Data source

Bangladesh Household Income and Expenditure Survey (HIES) 2010, conducted by Bangladesh Bureau of Statistics (BBS) [22] provides data for this study. The authors bought the data set from BBS agreeing the rules and regulations of data use.

Data were collected using a two-stage stratified random sampling technique under the framework of Integrated Multipurpose Sampling (IMPS) design. The design consisted of 1000 Primary Sampling Units (PSUs) throughout the country. There were 640 rural and 360 urban PSUs in the sample. The PSU was defined as contiguous two of more enumeration areas (EA) used in Population and Housing Census 2001. Each PSU comprised of around 200 households. In the first stage, 612 out of total 1000 IMPS PSUs, were drawn. These PSUs were selected from 16 different strata. There were 6 rural, 6 urban and 4 sub-urban municipal area (SMA) strata. In the second stage, 20 households were selected from each of the rural PSUs, and also PSUs located in the municipal areas and SMAs.

### Definitions of the key variables

Chronic illness: Chronic disease and chronic illness are used interchangeably. Diseases or illnesses that lasted or expected to last for 12 or more months are considered chronic illness/diseases. To understand the presence of chronic illness among the family members, the respondents were asked, “Have you or any member of your family suffered/suffering from any illness or disability lasting for the last 12 months or more?” Then, a list of chronic illness including cancer, diabetes, heart diseases etc. were mentioned. The duration of the specific disease was asked and recorded for cross checking.

Health Shocks: Presence of unpredictable illnesses among the family member(s) that diminish health status, and bring an economic burden to the family. To understand the presence of health shock in the family, the respondents were asked whether any household member faced any serious illness or injuries or death during the previous 12 months’ period. Further, the approximate date and duration were asked and recorded.

Out-of-pocket health spending: This is the share of the expenses that household pays directly to the healthcare provider without a third party. In our study, household health expenditure equals to household out-of-pocket spending on healthcare.

### Samples and variables

The HIES 2010 constitutes 12,240 households including 55,618 individuals. Among them, 10,701 (87 · 43%) households incurred healthcare expenditure during the previous 12 months period. The dependent variable is total household annual healthcare expenditure measured in Bangladesh currency Taka (Tk.) equivalent to U.S. $0 · 0128 (1 · 25 cents). The predictor variables are: yearly income, presence of chronic illness, health shocks, proportion of uneducated persons in the family, place of living, family size, proportion of household members who are females, under-five, and aged 60 and over.

The mean household annual healthcare expenditure was Tk. 782, whereas the median was Tk.200, with high skewness (71) and very abnormal kurtosis (6144). After log transformation, the mean becomes Tk.5 · 37 and the median is Tk. 5 · 30, skewedness is 0 · 27 and kurtosis is 3 · 16. Variables and their measurements are shown in Table 1. Mean yearly income of household was TK. 107,000 with a median of Tk.55, 000. The mean duration of chronic illness was 21 months. The average household size was 4 · 49 persons per family, with 49% females. Among the family members 10% were under five and 9% were 60 years and above. In respect of literacy, around 51% were uneducated and 49% were educated members in the family. Among those educated, 43% completed junior school, and about 6% completed college and above level education. Sixty-nine percent of the sampled households lived in rural areas, 26% suffered from chronic illnesses and around 4% of the households suffered from health shocks.

**Table 1:** Variables and measurements

Variables	Measurements	Mean	Max	Min	SD
Dependent variable					
Log of yearly total household healthcare expenditures (ltothhexp)	Log transformation of household yearly healthcare expenditure	5 · 37	13 · 13	0	1 · 47
Predictor variables					
Log of yearly total household income (ltothhyrinc)	Log transformation of household yearly income in 1000 Tk.	3 · 97	8 · 77	−2 · 99	1 · 31
Proportion of either illiterate or did not complete junior school in family (prilliterate)	Proportion of either illiterate or did not complete junior school in family (prilliterate)	0 · 51	1	0	0 · 31
Log of total household durable goods	Log of total household durable goods valued in Tk.	9 · 34	15 · 22	3 · 40	1 · 41
Family size (famsize)	Number of family members	4 · 49	17	1	1 · 83
Proportion of 60 and above aged members in family (prsenmem)	Proportion of family members aged 60 years and above	0 · 09	1	0	0 · 18
Proportion of under-five in family (prunder5)	Proportion of under five members in the family	0 · 10	0 · 67	0	0 · 13
Proportion of females in family (prfemales)	Proportion of female members in the family	0 · 49	1	0	0 · 19

### Statistical approach

The majority of the studies from the developing countries are descriptive in nature and very few took analytical approaches. The most used econometric techniques were ordinary least square (OLS) method. Few researchers used Tobit model [23]. However, Rous & Hotchkiss [24] suggested that the Tobit model should be applied carefully in the case of health expenditure. In Nepal, the researchers developed a full information maximum likelihood model to control endogeneity of sickness and provider choice [24]. In Zambia, a study validated the method to control endogeneity bias by generating selection term as a regressor in OLS estimation of healthcare expenditure for respective providers [25].

In choosing the analytical method, our goal was to use a simple, “begin-with” method that closely fit a function with the data by minimizing the sum of square errors. Our objective was to predict the household out-of-pocket healthcare expenditure on households’ other characteristics like proportions of females, under-fives, and senior members along with income and presence of chronic illnesses. Considering the fact that our data come from a multistage cluster sampling, we use survey linear regression. However, we have tested models adding interactions like, being rural and having chronic illness, total household healthcare expenditure, health shock, proportion of under-five children and senior members. All the models are non-significant except being rural inhabitant and total household healthcare expenditure have negligible positive coefficient (0.0001). Also, we have tested heteroscedasticity of the random variables as well as presence of multicollinearity.

**Fig. 1:**
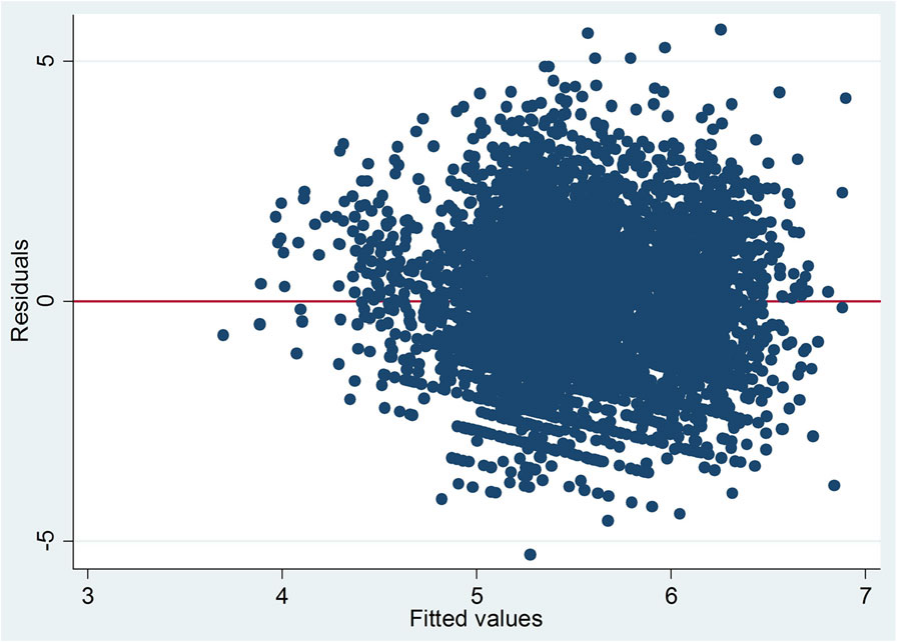
Homoscedasticity of the residuals

**Fig. 2:**
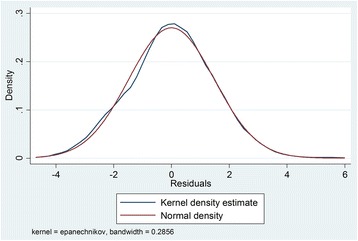
Normality of the residuals using Kernel density curve

Predictors of household healthcare expenditure were analyzed using survey linear regression. Statistical analyses were performed using STATA 14 · 0. Influential outliers were identified and deleted by examining the studentized residuals. The Breusch-Pagan/Cook-Weishberg test and the variance of inflation factors tests were conducted to determine heteroscedasticity (Fig. 1) and multicollinearity. Normality of the residuals was verified using Kernel density curve (Fig. 2). A *p*-value of 0 · 05 or less was adopted as the statistical significance level. As the data comes from a multi-stage clustering sample, the required survey adjustments were made to the standard estimation of OLS, so that estimation parameters and standard errors are valid under this complex survey scheme. The outcome variable, household healthcare expenditure and a predictor variable, household income and household durable goods were log transformed to satisfy the OLS assumptions, and to reducing the influence of outliers. Presence of chronic illness, health shocks and place of living are dichotomous. We model non-linear relationships using logarithmic form in a linear regression model. Therefore, our regression model is linear in parameters. In the empirical analysis, we specified the following model:

Y_i_ (log of household health expenditure) = β_0_ + β1chronic illness_i_ + β_2_log of household income_i_ + β_3_Health shocks_i_ + β_4_proportion of uneducated members_i_ + β_5_log of household durable goods_i_ + β_6_Family size_i_ + β_7_ Proportion of elderly_i_ + β_8_Proportion of under5_i_ + β_9_Proportion of female member_i_ + β_10_Place of living_i_ + ε_i_

Where; β_0_ = intercept; _i_ = family; and ε_i_ = error term.

## Results

After deletion of influential observations, the distribution of residuals becomes homoscedastic (Fig. 1) and almost normal (Fig. 2). In running the interaction models, we did not find so much changes in the model except interaction between being rural and total household healthcare expenditure. For the test of heteroscedasticity, we ran a graphical test of homoscedasticity of the residuals (Fig. 2). The errors are normally distributed, and the model has a well fit. The coefficients of VIF test is around 1.00, and we concluded that there is no harmful collinearity among the variables.

The results of multiple regression show that 10% increase in household income leads to a 2% increase in household healthcare expenditure holding all other variables constant in the model, and it is highly statistically significant at *p* < 0 · 001. Respective regression coefficients, linearized standard errors, *t*-value, significance with 95% confident intervals are presented in Table 2.

**Table 2:** Findings from multiple regression

Variables	OLS β-coefficient	Linearized standard error	*t*-value	Significant level *p* > |t|	95% Confidence Intervals	
					Lower value	Upper value
Presence of chronic illness	·70	·05	13 · 78	0 · 000^***^	·60	·81
log of total household income	·20	·02	9 · 88	0 · 000^***^	·16	·24
Presence of health shock	·30	·15	2 · 03	0 · 04^*^	·01	·60
Proportion of illiterate members in the family	– · 11	·08	−1 · 52	0 · 13	– · 26	·03
Log of household durable goods	·01	·02	0 · 78	0 · 44	– · 02	·05
Family size	·02	·01	2 · 22	0 · 03^*^	·00	·04
Proportion of members age 60 and above	·05	·11	0 · 49	0 · 63	– · 16	·27
Proportion of under-five children	·22	·16	1 · 38	0 · 17	– · 09	·54
Proportion of female members	– · 02	·11	−0 · 19	0 · 85	– · 24	·20
Rural residence	– · 07	·07	−0 · 94	0 · 35	– · 22	·08

***significant < 0 · 001 level; *significant <0 · 05 level

Presence of chronic illnesses in the household found to be statistically significant with household healthcare expenditure (*p* < 0 · 001). Having chronic illness among the household member leads to 101% increase in annual household healthcare expenditure in comparison to households without chronic illnesses. Health shocks were measured as having any accidental deaths or injuries. Health shocks in the household leads to 35% increase in household healthcare expenditure and found to be statistically significant.

The results on educational attainments show that 10% increase in uneducated members leads to a 11% decrease in household health expenditure holding all other variables in the model constant. Households with more uneducated^1^ members use less healthcare because they either are less knowledgeable about availability of healthcare, or have preference for home or alternative remedy. A 100% increase in female members in the household leads to a 2% decrease in healthcare expenditure. Though statistically insignificant, these findings have been checked with all regression models and found to be in the same direction of the coefficient.

We investigate the effects of proportion of under-five and members 60 and above years of age in the family. A 10% increase in under-five children in the family leads to a 2.2% increase in household healthcare expenditure, though statistically insignificant (*p* > 0 · 16). A similar finding was observed in households with members aged 60 and above. A 10% increase in aged members in the family leads to a 0.5% increase in household healthcare expenditure, though statistically insignificant. Both the findings are similar in nature and have an important policy implication, as the country is undergoing a rapid demographic transition. Another finding, not surprising though, of this study is that rural households spend 7% less on healthcare than their urban counterpart. Rural people are usually less educated and hence less health awareness and there are less healthcare services available in rural area. Moreover, they cannot afford specialist doctors/hospitals and sophisticated technology.

## Discussions

The aim of this study was to understand the comparative role of household income, presence of chronic illness and health shocks in the family on household healthcare expenditure. While such a topic has been extensively studied, the situation in Bangladesh have not been well documented. It is a well-known perception that household income is the strongest predictor of health expenditure. The findings of this study showed that besides income, there are other more important predictors when we control related household variables. These are chronic illness and health shocks in the family. These findings are consistent with the earlier studies by Hjortsberg [25] and Rous and Hotchkiss [24]. However, raising income is not the direct purview of health policy, though financial means are important for explaining amount of healthcare expenditure.

Presence of chronic illness in the household was found to be the most important predictor of households’ health expenditure. In case of chronic illness, doubling household health expenditure is consistent with other findings in the middle- and low-income countries and one study on Bangladesh population [16]. Bangladesh system of chronic care management is only limited to focusing on treating the patients without functional preventive and promotive measured of the conditions. As aging populations are increasing at a faster rate than before, it needs a comprehensive system of change including prevention and promotion [26].

Similar to illness, health shocks in the household is another important predictor of household healthcare expenditure. In case of health shocks households use income, savings, borrowing, loans or mortgages, selling assets and livestock to meet the stock [27]. Studies showed that developing health insurance scheme in case of health shocks helps households maintain financially stable [28]. In Bangladesh, both public and private health insurance coverage had been very low or even non-existing, and formal insurance and credit markets are also less developed. Therefore, it is recommended that health insurance in both sectors need to be developed/strengthened, if not for all cases, but at least for health shocks, such as catastrophic health insurance.

The results show that households with more proportion of uneducated members spend less on healthcare than households with more proportion of junior school completers. This supports the findings from Bangladesh that secondary school and higher educated people spend more on healthcare [16]. It means that households with uneducated members in the family spend less on healthcare than households with educated members. This is because they either are less knowledgeable about availability of healthcare, or have preference for home or alternative remedy. The educated members are more knowledgeable about healthcare availability and have preference for modern sophisticated medical care.

Another unexpected finding is that, 100% increase in female members in comparison to male members leads to a 2% decrease in household health expenditure that supports the findings of Akanda and Minowa [16] and Sarker et al. [29]. This seems to be paradoxical and appears to be opposite of what is seen in high-income countries. This signals gender discrimination of female households that resulted in same health conditions; male members are more likely to seek professional healthcare. Despite a dramatic increase on women employment in the last decade, women employment in Bangladesh is still as low as 26% [30]. In South Asia, women are seen as additional mouths, not additional hands. Additional socio-demographic factors, like differentials in sickness reporting as well as consulting, cultural and social factors led household resource allocation process to favor male than female [31]. A study from Bangladesh also supports that household health expenditure for males is proportionately more (US $11 · 5) than females (US $11 · 2) [29].

Population structure of the society exerts a great impact on health expenditure. This study includes population of under-five and above 60 years old. It is well-known that elderly population suffers more from chronic illnesses and requires more healthcare which results in a higher healthcare expenditure. Therefore, we recommend to launch especial insurance scheme for the elderly. It is expected that government would devote more domestic resources to lessen the burden of health expenditure in households with elderly members from being in poverty. Similarly, under-five children need to be covered by the public sector or in case of insufficiency of public funds, needs to arrange a safety net, or launch a separate insurance program for this population.

Despite the fact that survey is a dominant form of data collection in low-income countries, this study has some limitations. The survey relied on self-reporting and is prone to recall/reporting bias. We used household as a unit of analysis that does not account for any complexity of diversity of families. In addition, the data contain large number of zero OOP payments. This might be related with the fact that poor households did not use health services and could not make any payments at all, which needs further analysis and interpretation.

The findings of this study suggest that rural household on average spend 7% less on healthcare than their counterpart in urban area, controlling for income, education, and household size. This is related with the fact that modern medical facilities and specialists are mostly available in urban areas. These findings support the findings from Akanda and Minowa [16]. On the other hand, this finding contradicts those of Hotchkiss et al. [32].

## Conclusions

The results of this study showed that sickness is not the only the predictors of healthcare expenditure. The other influential predictors are presence of chronic illness, health shocks, place of living, proportion of female members, under-five and elderly. Together, all these factors predict the amount of household healthcare expenditure. A safety net needs to be provided or strengthened for low-income rural households and for elderly members. The most strategic and attainable programs would be the control and prevention of chronic diseases. Universal coverage of healthcare would be the final solution. After 2010, The Ministry of Health and Family Welfare have strengthened previous programs as well as launched some new types of safety net for the poor, the disabled and women. However, further strengthening is essential and hence recommended. Further research is warranted to understand the causes of gender disparity on household health expenditure. Alternate revenue generation and allocation of resources to cover the health needs of the people need to be revisited and relocated. For example, exemption process of fees for the poor, disabled and disadvantaged can alleviate their OOP healthcare expenditure and financial burden. As the government and the people of Bangladesh are concerned about the high OOP healthcare expenditure, our study suggests that the country needs to reform health system finance scheme.

## Abbreviations

BBS: Bangladesh Bureau of Statistics
ESP: Essential services package
GDP: Gross domestic product
HIES: Household income and expenditure survey
OOP: Out-of-pocket payment
PSU: Primary sampling unit
TK: Taka (Bangladesh currency)

## Footnotes

1 Household adult members either illiterate or not complete junior school.
